# Purification, Characterization, and Anti-Inflammatory Potential of Free and Bound Polyphenols Extracted from *Rosa roxburghii* Tratt Pomace

**DOI:** 10.3390/foods13132044

**Published:** 2024-06-27

**Authors:** Chao Li, Hengyi Li, Xiong Fu, Qiang Huang, Yinghua Li

**Affiliations:** 1 Guangdong Province Key Laboratory for Green Processing of Natural Products and Product Safety, School of Food Science and Engineering, South China University of Technology, Guangzhou 510640, China; felichao@scut.edu.cn (C.L.); czym333@163.com (H.L.); lfxfu@scut.edu.cn (X.F.);; 2 Center Laboratory, Guangzhou Women and Children’s Medical Center, Guangzhou Medical University, Guangzhou 510120, China

**Keywords:** polyphenols, purification, anti-inflammatory, antioxidant, mechanism

## Abstract

*Rosa roxburghii* Tratt pomace (RRTP), an underutilized byproduct, is rich in polyphenol compounds. This study aimed to further explore the purification, characterization, anti-inflammatory activities, and underlying molecular mechanisms of free polyphenols (RRTP-FP) and bound polyphenols (RRTP-BP) from RRTP. The results indicated that AB-8 macroporous resin emerged as the preferred choice for subsequent separation and purification. The purities of purified RRTP-FP (P-RRTP-FP) and purified RRTP-BP (P-RRTP-BP) increased by 103.34% and 66.01%, respectively. Quantitative analysis identified epigallocatechin, epicatechin, and ellagic acid as the main phenolic compounds in P-RRTP-FP. In P-RRTP-BP, the primary phenolic compounds were ellagic acid, epicatechin, and gallic acid. In vitro antioxidant assays demonstrated the superior DPPH and ABTS radical scavenging activities of P-RRTP-FP and P-RRTP-BP compared to vitamin C. Treatment with P-RRTP-FP and P-RRTP-BP reduced nitric oxide (NO) and reactive oxygen species (ROS) production, mitigated the decline in cellular membrane potential, and significantly downregulated the mRNA expression of pro-inflammatory cytokines and inducible nitric oxide synthase (iNOS) in lipopolysaccharide (LPS)-stimulated RAW264.7 macrophages. Additionally, P-RRTP-FP and P-RRTP-BP inhibited the phosphorylation of pertinent proteins in the nuclear factor kappa-B (NF-κB) and mitogen-activated protein kinase (MAPK) signaling pathways. This finding suggests potential utility of RRTP-derived polyphenols as anti-inflammatory agents for managing severe inflammatory conditions.

## 1. Introduction

Inflammation, a pivotal immune response to infection or irritation, plays a crucial role in eliminating pathogens and promoting tissue repair. However, dysregulated or chronic inflammation is implicated in various pathological conditions, including asthma, cardiovascular diseases, cancer, and neurodegenerative diseases [1]. While conventional anti-inflammatory drugs like non-steroidal anti-inflammatory drugs (NSAIDs) and glucocorticoids (GCs) offer symptomatic relief, their adverse effects on the cardiovascular system, with the exception of aspirin, underscore the need for safer alternatives. Prolonged use of these medications can also precipitate gastrointestinal bleeding and organ damage, necessitating exploration of natural compounds with fewer side effects [2]. Polyphenols derived from natural sources have emerged as promising candidates for managing inflammation due to their potent anti-inflammatory properties and relatively low toxicity profile [3,4,5,6].

*Rosa roxburghii* Tratt (RRT), commonly known as ‘Cili’ in China, is a plant resource that is widely distributed in the southwest of China, renowned for its high nutritional value and health benefits. Recent studies have identified numerous bioactive components in RRT, such as organic acids, phenols, flavonoids, polysaccharides, triterpenoids, and vitamins [7]. In recent years, various food products derived from RRT fruit have been developed, such as tea, yogurt, vinegar, jam, preserved fruit, and cake. While most of these products utilize RRT juice or whole fruit as raw materials, a significant amount of RRT pomace (RRTP) is generated as a byproduct during juice extraction, often discarded despite containing valuable resources [8,9]. Our previous study revealed that RRTP is rich in phenolic compounds, and the extracts of both free polyphenols (RRTP-FP) and bound polyphenols (RRTP-BP) from RRTP exhibited remarkable antioxidant properties in vitro and in vivo [10]. However, the raw extracts contain impurities, such as pigments, polysaccharides, and proteins, which exhibit dissimilarities from the polyphenols in regards to their composition, content, and functional properties. Macroporous resins provide a cost-effective and environmentally friendly method for purifying polyphenols, making them suitable for large-scale industrial production [11]. For instance, NKA-9 resin effectively enriched bound polyphenols from mung bean coat dietary fiber, resulting in a product with enhanced antioxidant activity [12]. Similarly, purification with AB-8 macroporous resin increased the antioxidant activity of sweet potato leaf polyphenols significantly compared to grape seed polyphenols [13].

Therefore, the aim of this study was to purify RRTP-FP and RRTP-BP using macroporous resins, and then to systematically investigate the composition of purified RRTP-FP (P-RRTP-FP) and RRTP-BP (P-RRTP-BP), as well as their in vitro antioxidant and anti-inflammatory activities. Quantitative analysis of P-RRTP-FP and P-RRTP-BP was conducted using high-performance liquid chromatography (HPLC). The in vitro antioxidant activities of P-RRTP-FP and P-RRTP-BP were evaluated using DPPH, ABTS, and reactive oxygen species (ROS) assays. Anti-inflammatory assessment was performed using RAW264.7 macrophage cells induced by lipopolysaccharide (LPS). This study will provide a theoretical basis for the utilization of RRTP as a natural anti-inflammatory agent in the food industry, contributing to the development of functional foods with enhanced health benefits.

## 2. Materials and Methods

### 2.1. Materials

*Rosa roxburghii* Tratt pomace (RRTP) was kindly provided by Guizhou Xinyang Agricultural Science and Technology Development Co. Ltd. (Bijie, Guizhou, China). RRTP is the residue remaining after extracting juice from *Rosa roxburghii* fruit by squeezing. Upon receipt, RRTP underwent drying in an air-drying oven at 45 °C for 48 h and was then stored under sealed conditions at room temperature. 1-Diphenyl-2-picrylhydrazyl (DPPH), 2,2′-amino-di (2-ethyl-benzothiazoline sulfonic acid-6) ammonium salt (ABTS) and reference standards were purchased from Sigma-Aldrich Chemical Co. (St Louis, MO, USA). Other solvents used for HPLC analysis were of chromatographic grade. The macroporous resins (DM130/AB-8/D101) were bought from Shanghai Yuanye Bio-Technology Co., Ltd. (Shanghai, China). Dulbecco’s modified Eagle’s medium (DMEM), fetal bovine serum (FBS), streptomycin, and penicillin were purchased from Gibco Life Technologies (Grand Island, NY, USA). Cell Counting Kit-8 (CCK-8), mitochondrial membrane potential assay kit with JC-1 (JC-1), and reactive oxygen species assay kit (ROS) were purchased from Beyotime Biotechnology (Shanghai, China).

### 2.2. Extraction of Free and Bound Polyphenolic Fractions from RRTP

The extraction of RRTP-FP from RRTP was conducted using a solvent extraction method [10]. Initially, dried RRTP with the seeds and impurities removed was pulverized using a laboratory mill (FW 135, Taisite, Tianjing, China) and then sieved through a 60-mesh sieve to obtain a fine powder. Then, the RRTP powder underwent defatting with n-hexane three times. The resulting residue was collected via centrifugation at 5000× *g* for 10 min and subsequently dried at 45 °C in an air-drying oven. The defatted sample (5 g) was then suspended in 75 mL of 70% ethanol and continuously stirred at room temperature for 2 h. The supernatant was isolated by centrifugation at 8000× *g* for 10 min. This process was repeated twice with the residue. The three supernatants were combined and subsequently lyophilized to yield the RRTP-FP.

During the extraction of RRTP-BP, alkaline hydrolysis was employed due to its binding to dietary fiber via both covalent and non-covalent bonds [14,15]. The residue remaining after the extraction of RRTP-FP was mixed with NaOH (4 mol/L) at a ratio of 1:10 g/mL and stirred for 2 h under an oxygen-free atmosphere. Following this, the pH of the solution was adjusted to 2.0 using HCl solution (6 mol/L). Subsequently, the mixture underwent six extractions with ethyl acetate. The resulting organic fractions were gathered, concentrated, and dried to yield the RRTP-BP.

### 2.3. Purification of RRTP-FP and P-RRTP-BP by Macroporous Resins

#### 2.3.1. Screening of Macroporous Resins

The screening process was operated as described in a published report, with slight modifications [16]. For the purpose of screening the optimal resin, the static adsorption capacity, adsorption rate, and desorption rate of three types of macroporous resins (DM130/AB-8/D101) were determined.

(1) $$\mathrm{Adsorption}\;\mathrm{rate}\;\left(\%\right)=\frac{C_{0}V_{0}-C_{1}V_{1}}{C_{0}V_{0}}\times 100$$

(2) $$\mathrm{Desorption}\;\mathrm{rate}\;\left(\%\right)=\frac{C_{2}V_{2}}{C_{0}V_{0}-C_{1}V_{1}}\times 100$$

(3) $$\mathrm{Adsorption}\;\mathrm{capacity}\;\left(\mathrm{mg}/g\right)=\frac{C_{0}-C_{1}}{M}\times V_{0}$$

where M is the mass of resin in the conical flask (g), C_0_ is the concentration of total polyphenols in the crude extract of RRTP (mg/mL), C_1_ is the concentration of total polyphenols in the conical flask at equilibrium (mg/mL), C_2_ is the concentration of total polyphenols in the eluent solution (mg/mL), V_0_ is the volume of the RRTP extract solution added during measurement (mL), V_1_ is the volume of the filtrate after adsorption equilibrium (mL), and V_2_ is the volume of the eluent solution (mL).

#### 2.3.2. The Adsorption Kinetics of AB-8 Resin

Briefly, a sample solution (1 mg/mL, 25 mL) was mixed with 2 g of macroporous resin and shaken at 28 °C. The total phenol content was determined from the supernatant at hourly intervals to investigate the adsorption kinetics of AB-8 resin, representing the variation in adsorption capacity over time under constant temperature conditions. The adsorption kinetics of the resin was analyzed using pseudo-first-order, pseudo-second-order, and intra-particle diffusion kinetics models:

(4) $$Pseudo\text{-}first\text{-}order\;model\;equation:\;\ln(q_{e}-q_{t})=-k_{1}t+\ln q_{e}$$

(5) $$Pseudo\text{-}second\text{-}order\;model\;equation:\;t/q_{t}=1/k_{2}q_{e}^{2}+t/q_{e}$$

(6) $${Intra\text{-}particle\;diffusion\;model\;equation:\;q}_{t}=k_{p}t^{1/2}+C$$

where q_t_ (mg/g) represents the adsorption amount at time t (h); k_1_ (h^−1^), k_2_ [g/(mg·h)], and kp [mg/(g·h^1/2^)] represent the rate constants of the pseudo-first-order, pseudo-second-order, and intra-particle diffusion models, respectively; and C (mg/g) represents a constant dependent on the boundary layer thickness.

#### 2.3.3. Static Adsorption and Desorption Assay

The static adsorption and desorption was carried out according to a portion of the methodology based on the previous approach [16]. Briefly, samples of solutions at different pH values (2, 3, 4, 5, 6, and 7) with a concentration of 1 mg/mL and a volume of 25 mL were mixed with 2 g of macroporous resin and shaken at 28 °C for 24 h. The supernatant was then collected to determine the total phenol content and calculate the adsorption rate. Following the completion of the adsorption process, the macroporous resin was filtered and rinsed with deionized water. Subsequently, the resin was treated with an ethanol solution (90%, 25 mL), and static desorption kinetics curves were constructed based on time and desorption capacity.

#### 2.3.4. Dynamic Adsorption and Resolution Assay

Various parameters, including the loading flow rate, loading volume, eluent volume fraction, elution flow rate, and elution volume, were investigated to determine their effects on the elution process. The selected optimal resin was wet-loaded into a 1.6 cm × 20 cm glass chromatography column with a column bed volume (1 BV) of 30 mL. The crude RRTP-FP and RRTP-BP extracts (1 mg/mL) were then adsorbed on the column at loading flow rates of 1.0, 1.5, and 2.0 mL/min, with the leachate collected in 5 mL fractions. The point at which the polyphenol concentration in the leachate reached one-tenth of the original sample was considered to be the leakage point, determining the optimal loading flow rate and volume.

Subsequently, the sample was loaded under the optimal loading flow rate and volume, and 3 BV of leachate was collected using ethanol as an eluent, with volume fractions of 50%, 60%, 70%, 80%, and 90%. Desorption rates were calculated to determine the optimal eluent concentration.

After fixing the optimal loading conditions and eluent volume fraction, elution proceeded at flow rates of 1.0, 1.5, and 2.0 mL/min, with 5 mL fractions collected for a total of 3 BV. Elution curves were plotted at different elution rates to determine the optimal elution flow rate and volume.

### 2.4. Determination of Total Phenolic Contents

The total phenolic content (TPC) of the tested samples was measured using the Folin–Ciocâlteu reagent method. The absorbance at 760 nm was determined using a microplate reader (BioTek, Winooski, VT, USA), and the TPC was quantified as milligrams of gallic acid equivalents (GAE) per gram of extract (mg GAE/g E).

### 2.5. Quantification of the Contents of Individual Phenolic Compounds

The quantification of individual phenolic compounds was analyzed according to our previously established method [10]. The contents of RRTP-FP, P-RRTP-FP, RRTP-BP, and P-RRTP-BP were quantified using an Agilent Series 1260 system equipped with a diode array detector (DAD) and auto-sampler. A ZORBAX SB-C18 (4.6 × 250 mm, 5 μm) column was adapted, with the column temperature set at 30 °C. The mobile phases contained 0.1% formic acid in aqueous solution (solution A) and acetonitrile (solution B) at a flow rate of 0.8 mL/min. Samples were filtered through a 0.22 μm membrane filter prior to analysis, with a flow rate and injection volume of 0.8 mL/min and 10 μL, respectively. Based on our previous research, the main components of RRTP-FP and RRTP-BP include gallic acid, ellagic acid (EA), epigallocatechin, epicatechin, and quercetin [10]. Therefore, these six polyphenolic compounds were used as standards. The content of each polyphenolic compound was expressed as milligrams of standard per gram of extract (mg/g).

### 2.6. In Vitro Antioxidant Assay

#### 2.6.1. DPPH Radical Scavenging Activity

The DPPH radical scavenging activity was evaluated according to the method previously described in [10]. The absorbance was measured at 517 nm. Ascorbic acid (VC) was used as a positive control, and 70% methanol solution was used as a blank. The DPPH radical scavenging rate was calculated using the following formula:

(7) $$Scavenging\;rate(\%)=\left(1-\frac{A_{s}-A_{b}}{A_{c}}\right)\times 100$$

where A_S_ represents the absorbance value of the reaction system containing the sample and DPPH solution, while A_b_ and A_C_ denote the absorbance values of the reaction systems without DPPH solution and sample, respectively.

#### 2.6.2. ABTS Radical Scavenging Activity

The ABTS radical scavenging activity was evaluated according to the method previously described in [10]. Absorbance was measured at 734 nm, and the ABTS radical scavenging activity was calculated using the following formula:

(8) $$Scavenging\;rate\left(\%\right)=\left(\frac{A_{0}-A_{1}}{A_{0}}\right)\times 100$$

where A_0_ and A_1_ represent the absorbance values of the blank reaction system and the sample reaction system, respectively.

### 2.7. Cell Culture

The murine macrophage cell line RAW264.7 was supplied by the Center of Cellular Resources (Chinese Academy of Sciences, Shanghai, China). Cells were cultured in DMEM supplemented with 10% fetal bovine serum (FBS) and 1% penicillin and streptomycin (PS) in a humidified incubator at 37 °C and 5% CO_2_.

### 2.8. Cell Viability Assay

Cell viability was evaluated using the Cell Counting Kit-8 assay. RAW264.7 cells were seeded into 96-well plates at a density of 1.8 × 10^5^ cells/mL, with six parallels in each group. After 24 h, the blank and control groups were treated with 100 µL of complete medium, while the experimental group was treated with 100 µL of varying concentrations of P-RRTP-FP and P-RRTP-BP. After 24 h of induction, the CCK8 solution (complete medium: CCK8 solution = 10:1) was replaced in each well for the reaction. The absorbance of the cell plates was measured at 570 nm using an enzyme labeler, and cell viability was calculated according to the following formula:

Cell viability (%) = (OD_experimental_ − OD_blank_)/(OD_control_ − OD_blank_) × 100 (9)

### 2.9. Measurement of Nitric Oxide (NO) Production

RAW264.7 cells were seeded at a density of 1.8 × 10^5^ cells/well in 24-well plates and incubated for 24 h. Prior to treatment with 1 μg/mL LPS, the cells were pre-incubated with the samples for 8 h, followed by further incubation for 24 h. NO production was assessed using the Griess assay [17]. The absorbance at 540 nm was measured on a microplate reader (Multiskan GO, Thermo Fisher, Waltham, MA, USA).

### 2.10. Measurement of Intracellular ROS

Intracellular ROS release was measured by detecting the fluorescence intensity of the oxidant-sensitive probe 2′,7′-dichlorfluorescein-diacetate (DCFH-DA). RAW264.7 cells were seeded at a density of 2 × 10^5^ cells/well in 12-well culture plates and treated with different concentrations of P-RRTP-FP and P-RRTP-BP (50 and 100 μg/mL) for 8 h prior to stimulation with LPS (1 μg/mL) for 24 h. A portion of the cells was examined using a fluorescence microscope (Leica DMIL LED, Leica, Wetzlar, Germany), while another portion was collected for analysis using a fluorescence microplate reader (Multiskan GO, Thermo Fisher, Waltham, MA, USA). Excitation was performed at a wavelength of 488 nm, and emission was detected at 525 nm.

### 2.11. Determination of the Mitochondrial Membrane Potential

RAW264.7 cells were cultivated in a 12-well plate overnight, with a seeding density of 1.8 × 10^5^ cells per well. After treatment with P-RRTP-FP and P-RRTP-BP for 8 h, LPS (1 μg/mL) was added and incubated for 24 h. The cells were washed three times with PBS, and then 100 μL of JC-1 staining solution was added to each well. After incubation at 37 °C for 20 min, the cells were washed twice with pre-chilled JC-1 buffer to remove excess substrate. The fluorescence values of intact JC-1 monomers and JC-1 aggregates in mitochondria were measured using filters with wavelengths of 530 nm/590 nm and 485 nm/538 nm, respectively.

(10) $$Relative\;membrane\;potential\left(\%\right)=\frac{{\mathrm{OD}}_{\mathrm{aggregates}}-{\mathrm{OD}}_{\mathrm{blank}}}{{\mathrm{OD}}_{\mathrm{monomers}}-{\mathrm{OD}}_{\mathrm{blank}}}\times 100$$

### 2.12. Quantitative Real-Time PCR (qRT-PCR)

Total RNA was extracted from cells using TRIzol reagent, followed by cDNA synthesis through reverse transcription using a 7500 fluorescence quantitative PCR instrument (ABI, Los Angeles, CA, USA). Subsequently, qRT-PCR was conducted on the same instrument, employing SG Fast qPCR Master Mix (High Rox, B639273, BBI, Shanghai, China) along with specific primers. Relative gene expression (RFR) was determined using the comparative Ct method (RFR = 2^−ΔΔCT^), with GAPDH serving as the housekeeping gene. Primer sequences for analyzing TNF-α, IL-1β, iNOS, and GAPDH mRNA can be found in Table S1.

### 2.13. Western Blot Analysis

Protein concentrations were assessed using the BCA protein kit (Solarbio Science, Beijing, China). Following this, the proteins were separated through SDS-PAGE gel electrophoresis and transferred onto a PVDF membrane using a WB electrotransfer device. Subsequently, the PVDF membrane was sealed with Quick block solution (Beyotime Biotechnology, Haimen, China) for 10 min at room temperature. The primary antibody was then incubated at room temperature for 1 h, followed by overnight incubation at 4 °C. After washing off the primary antibody with TBST, the secondary antibody was applied and left to incubate for 2 h at room temperature. Protein bands were visualized using an enhanced chemiluminescence detection system (BIO-RAD, Hercules, CA, USA). GAPDH was employed as an internal reference, and the respective protein expression levels were quantified utilizing Image J software (version 1.53c).

### 2.14. Statistical Analysis

The results were presented as means ± SD. Univariate ANOVA was conducted using SPSS (version 20.0, IBM, Austin, TX, USA). ANOVA with Tukey’s test was used to compare the LPS group to the P-RRTP-FP and P-RRTP-BP groups, with significance set at *p* < 0.05.

## 3. Results and Discussion

### 3.1. Static Adsorption and Desorption

#### 3.1.1. Screening of Macroporous Resins

Different macroporous resins exhibit distinct adsorption and desorption characteristics with respect to natural products, owing to their unique polarity, specific pore sizes, and surface areas. To identify the most suitable resin for efficient enrichment of RRTP-FP and RRTP-BP, the adsorption and desorption capabilities of three resins were assessed and compared. As shown in Table 1 and Table 2, AB-8 macroporous resin displayed the best performance, likely due to its larger pore size and weak polarity, which facilitated the adsorption of polyphenols. Although DM130 resin with weak polarity has a high specific surface area, its adsorption and desorption capacities were slightly inferior. This suggests that appropriate polarity and specific surface area are key factors in purification [16]. Based on these findings, AB-8 macroporous resin was selected for further purification of RRTP-FP and RRTP-BP.

#### 3.1.2. Adsorption Kinetics of RRTP-FP and RRTP-BP on AB-8 Resin

Adsorption kinetics describes the rate at which an adsorbate is adsorbed by the adsorbent, controlled by the contact time of the adsorption reaction, which is an important characteristic defining adsorption efficiency [18]. The adsorption of polyphenols rapidly increases within the initial two hours, followed by a slowing down, ultimately reaching equilibrium at approximately 5 h. To evaluate adsorption kinetics data, various models such as the pseudo-first-order, pseudo-second-order, and Weber and Morris intra-particle diffusion models are employed.

**Figure 1:**
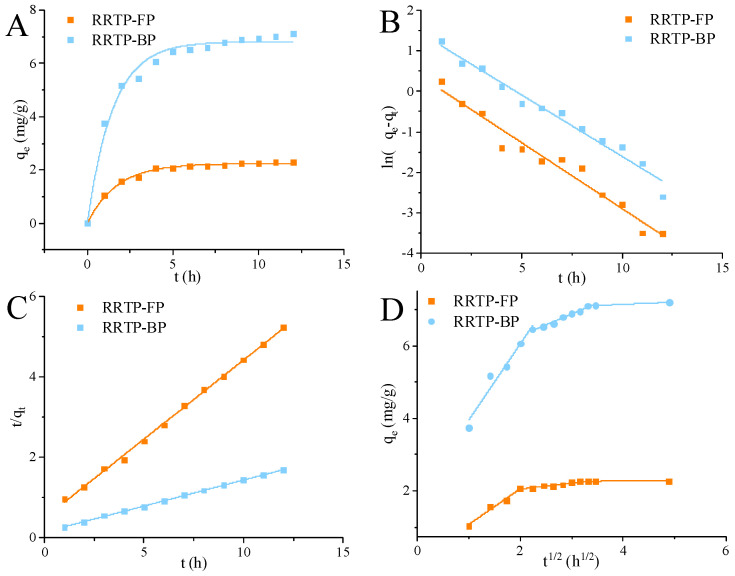
(**A**) Adsorption kinetics curve, (**B**) pseudo−first−order, (**C**) pseudo−second−order, and (**D**) intra−particle diffusion models for purifying RRTP−FP and RRTP−BP on the AB−8 resin at 28 °C.

The fitting parameters from Figure 1, as well as Tables S2 and S3, indicate that the adsorption kinetics data of AB-8 on RRTP-FP and RRTP-BP conform to the pseudo-second-order kinetics model, with the highest linear correlation coefficients of 0.998 and 0.999, respectively. The theoretical adsorption capacities (q_e_ = 2.207 mg/g for RRTP-FP and q_e_ = 7.078 mg/g for RRTP-BP) derived from the pseudo-second-order kinetics model align closely with the experimental data (2.33 mg/g for RRTP-FP and 6.67 mg/g for RRTP-BP).

Hence, the pseudo−second−order kinetics model provides a more accurate description of the mechanism involved in the purification of RRTP−FP and RRTP−BP by AB−8 macroporous resin. This suggests that the adsorption process of AB−8 macroporous resin encompasses a multi−layered and complex adsorption mechanism [19].

#### 3.1.3. Static Adsorption and Desorption Assay

**Figure 2:**
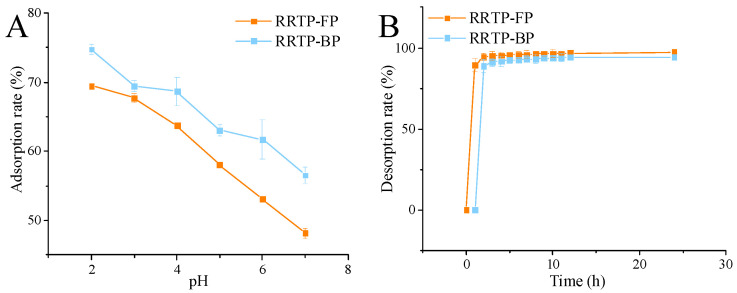
(**A**) Effect of pH on AB−8’s adsorption of RRTP−FP and RRTP−BP. (**B**) Static desorption curves of RRTP−FP and RRTP−BP. Data are shown as the mean ± standard deviation (*n* = 3).

The effect of the pH of the sample solution on the adsorption of AB−8 resin was investigated. As shown in Figure 2A,B, under acidic conditions, the adsorption rates of RRTP−FP and RRTP−BP decreased with increasing pH. Polyphenols are a class of compounds that contain several hydroxyl groups (−OH) and derive their polarity mainly from these hydroxyl groups, which may change under acidic conditions. Under acidic conditions, polyphenols exist in molecular form and are more easily adsorbed by macroporous resins. Higher pH leads to phenolic hydroxyl group dissociation, weakening hydrogen bonding and reducing the adsorption capacity [20]. Therefore, a pH value of 2 was selected as the optimal pH.

To further explore the purification mechanism of AB−8 resin and understand the dynamics of desorption over time, desorption kinetics curves were plotted. The desorption rates of RRTP−FP and RRTP−BP rapidly reached equilibrium within 1 h, with the desorption rates of the resin measured at 96.94% and 91.23%, respectively (Figure 2B). These findings indicate that AB−8 resin is capable of efficiently desorbing RRTP−FP and RRTP−BP with high recovery rates.

### 3.2. Dynamic Adsorption and Desorption of AB−8 Resin

#### 3.2.1. Effects of Loading Flow Rate and Loading Volume

**Figure 3:**
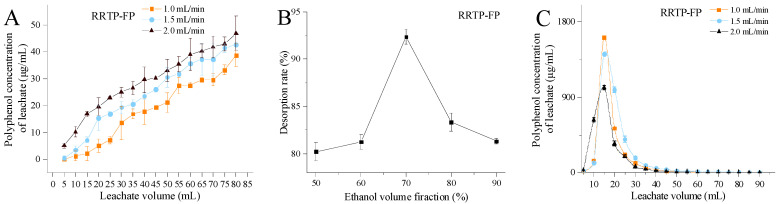

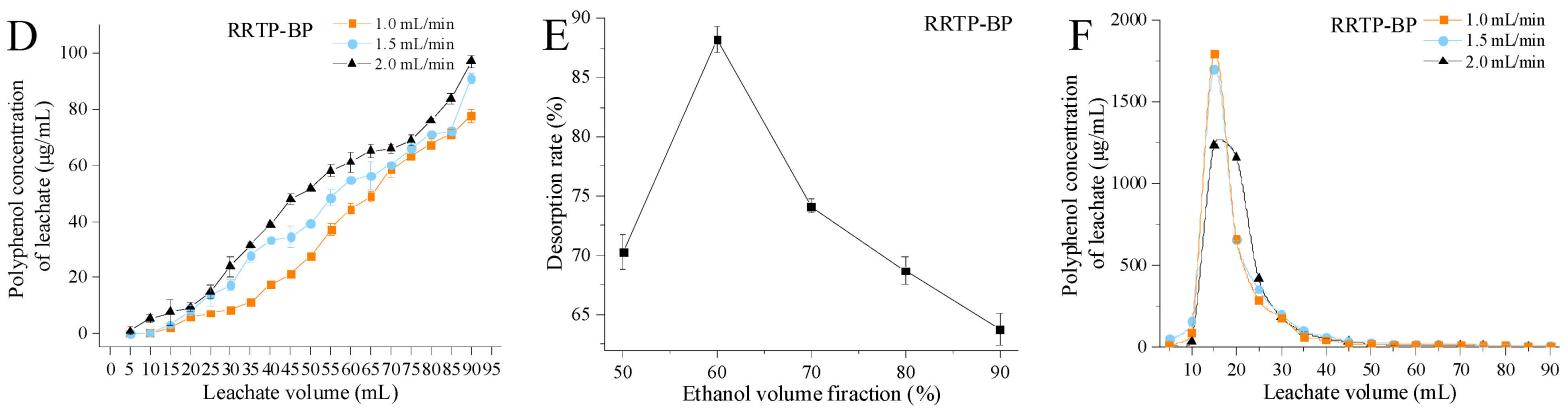
Effects of various factors on dynamic adsorption and desorption processes: (**A**,**D**) The impact of loading flow rate and loading amount on the adsorption capacity. (**B**,**E**) The effect of eluent volume fraction on the desorption capacity. (**C**,**F**) The impact of elution flow rate on desorption capacity. Data are shown as the mean ± standard deviation (*n* = 3).

The effects of the loading flow rate and loading volume on the adsorption of phenolic compounds were investigated, with the breakthrough curve serving as a crucial indicator for determining the operation and dynamic response of an adsorption column. Typically, the breakthrough point, defined as the point at which the outlet solution’s concentration reaches approximately 10% of the inlet solution, marks the completion of the adsorption process in industrial settings. As shown in Figure 3A, the breakthrough point of RRTP−FP occurred at approximately 50 mL (1.7 BV), 35 mL (1.2 BV), and 25 mL (0.8 BV) at flow rates of 1.0, 1.5, and 2.0 mL/min, respectively. Similarly, as shown in Figure 3D, the breakthrough point of RRTP−BP was observed around 65 mL (2.2 BV), 55 mL (1.8 BV), and 45 mL (1.5 BV) at the corresponding flow rates. As the flow rate increased, the breakthrough point occurred earlier. This phenomenon can be explained by the mass transfer mechanism of macroporous resin, where an increase in flow rate leads to a shorter contact time between the polyphenols and resin, resulting in incomplete adsorption [12]. Polyphenols could achieve full contact with resin at a low flow rate and show a satisfactory adsorption effect [21]. However, excessively slow flow rates could prolong the adsorption process and reduce the purification efficiency due to repeated adsorption of polyphenols. On balance, a loading flow rate of 1.5 mL/min was chosen as appropriate.

#### 3.2.2. Effect of Eluent Volume Fraction

In this study, 3 BV of ethanol with concentrations ranging from 50% to 90% was used to elute polyphenols from the AB−8 resin column at a flow rate of 1.5 mL/min to achieve complete desorption. The optimal eluent concentrations for RRTP−FP and RRTP−BP were determined to be 70% (Figure 3B) and 60% (Figure 3E), respectively, with maximum elution rates of 92.36% and 88.18%, respectively. This outcome can be attributed to van der Waals forces and hydrogen bonding between the polyphenols and resin. Low ethanol concentrations, being highly polar, hinder polyphenols’ dissolution. Conversely, high concentrations of less−polar ethanol may increase competitive dissolution of alcohol−soluble impurities, impacting polyphenols’ dissolution [21].

#### 3.2.3. Effects of Elution Flow Rate and Elution Volume

The desorption effects at various elution flow rates are shown in Figure 3C,F. Initially, the polyphenol content of the eluate increased rapidly upon the eluent’s addition. After reaching its peak, it gradually declined until nearly zero, as indicated thorough polyphenol extraction. At eluent flow rates of 1.0, 1.5, and 2.0 mL/min, the elution curves exhibited relatively concentrated and symmetrical peak shapes, suggesting effective elution of the AB−8 macroporous resin. Increased elution flow rates reduce desorption rates due to shorter contact time with the resin surface [22]. Conversely, slower elution prolongs the time passing through the chromatographic column, potentially causing polyphenol re−adsorption [12]. Based on these findings, a flow rate of 1.5 mL/min for elution was considered optimal, resulting in an elution volume of 60 mL (2 BV).

### 3.3. Total Phenolic Contents of RRTP−FP, P−RRTP−FP, RRTP−BP, and P−RRTP−BP

As shown in Table S4, the total phenolic contents of RRTP−FP and RRTP−BP were 213.33 and 501.86 mg GAE/g E, respectively, higher than that of pineapple pomace extract [23]. After purification, the total phenolic contents of P−RRTP−FP and P−RRTP−BP significantly increased to 433.79 mg GAE/g E and 833.11 mg GAE/g E, respectively, representing 2.35−fold and 1.66−fold increases compared to their respective unpurified forms.

### 3.4. Quantitative Analysis of Free and Bound Phenols by HPLC

The contents of RRTP−FP, P−RRTP−FP, RRTP−BP, and P−RRTP−BP were determined by HPLC, building upon previous research [10]. The quantitative analysis included six major components, including three phenolic acids and three flavonoids, as detailed in Table 3.

#### 3.4.1. HPLC Analysis of RRTP−FP and P−RRTP−FP

Phenolic acids in RRTP−FP are noteworthy. The main phenolic acid in RRTP−FP was protocatechuic acid, with the highest content of 2.4 mg/g E, followed by gallic acid (1.42 mg/g E) and ellagic acid (0.92 mg/g E). Meanwhile, RRTP−FP was also rich in flavonoids, with epigallocatechin being the most abundant, at 11.9 mg/g E, followed by quercetin (1.37 mg/g E), and epicatechin (0.77 mg/g E). It is worth noting that the AB−8 macroporous resin, being weakly polar, favored the enrichment of flavonoids over phenolic acids due to their lower polarity. P−RRTP−FP contained higher levels of flavonoids, specifically epigallocatechin and epicatechin, at 160.91 mg/g E and 31.96 mg/g E, respectively. These concentrations were 13.5 and 41.5 times higher than before purification, respectively. Additionally, the phenolic acid compound protocatechuic acid increased by 6.25 times compared to before purification, reaching 15 mg/g E.

#### 3.4.2. HPLC Analysis of RRTP−BP and P−RRTP−BP

For phenolic acid compounds, unlike RRTP−FP, the highest content in RRTP−BP was ellagic acid, at 23.38 mg/g E, followed by gallic acid (17.9 mg/g E). For flavonoids, the most abundant in RRTP−BP was epicatechin (7.45 mg/g E), followed by epigallocatechin (4.36 mg/g E) and quercetin (1.35 mg/g E). After purification with AB−8 macroporous resin, the contents of protocatechuic acid, ellagic acid, epicatechin, and quercetin in P−RRTP−BP were 14.8 mg/g E, 86 mg/g E, 63.27 mg/g E, and 14.03 mg/g E, respectively. This represents increases of 7.83 times, 3.67 times, 8.49 times, and 10.39 times higher than before purification, respectively.

### 3.5. Antioxidant Activity of P−RRTP−FP and P−RRTP−BP

**Figure 4:**
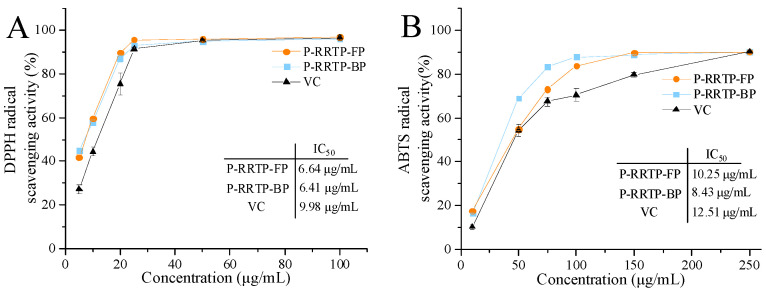
Antioxidant activities of P−RRTP−FP and P−RRTP−BP: (**A**) DPPH free radical scavenging activity. (**B**) ABTS free radical scavenging activity. Data are shown as the mean ± standard deviation (*n* = 3).

DPPH reagent possesses free radicals with unpaired electrons, exhibiting potent oxidative properties. This characteristic makes it widely used in vitro for measuring antioxidant activity. The IC_50_ values for P−RRTP−FP, P−RRTP−BP, and VC were determined to be 6.64 μg/mL, 6.41 μg/mL, and 9.98 μg/mL, respectively (Figure 4A). The purified products showed better ability to scavenge DPPH^•^ free radicals compared to VC.

The ABTS^•+^ assay offers the advantage of quantifying antioxidant substances that are soluble in both water and fat, and it is commonly used to evaluate antioxidant activity. The IC_50_ values for P−RRTP−FP, P−RRTP−BP, and VC were determined to be 10.25 μg/mL, 8.43 μg/mL, and 12.51 μg/mL, respectively (Figure 4B). The purified products showed better ability to scavenge ABTS^•+^ free radicals compared to VC.

Overall, both P−RRTP−FP and P−RRTP−BP exhibited significant antioxidant capabilities. This could be attributed to the chemical structures of the purified components [24]. Additionally, the enrichment of polyphenol contents through purification led us to speculate that different polyphenolic components in the purified product, such as EA and epigallocatechin, might synergistically enhance antioxidant activity.

### 3.6. Anti−Inflammatory Activities of P−RRTP−FP and P−RRTP−BP

#### 3.6.1. Effects of P−RRTP−FP and P−RRTP−BP on the Viability of RAW264.7 Cells

**Figure 5:**
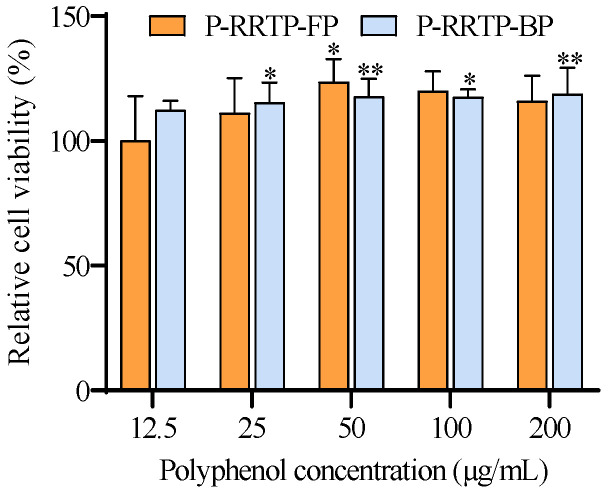
The impact of P−RRTP−FP and P−RRTP−BP on the viability of RAW264.7 cells; * *p* < 0.05 and ** *p* < 0.01 compared to the control. Data are shown as the mean ± standard deviation (*n* = 6).

Macrophages play a key role in the anti−inflammatory process [25]. The CCK−8 assay was utilized to assess the potential toxicity of P−RRTP−FP and P−RRTP−BP to the RAW264.7 cells. At low concentrations ranging from 15 to 50 μg/mL, compared to the control group, cell viability in the polyphenol−treated group increased with increasing polyphenol concentration (Figure 5).

Interestingly, when the concentration of P−RRTP−FP and P−RRTP−BP reached 50 μg/mL, the cellular viability peaked at 123.55% and 120.61%, respectively, compared to the control group. However, as the concentration of polyphenols increased from 50 to 200 μg/mL, cellular viability gradually declined. A previous study also observed a similar phenomenon: when polyphenols extracted from Chaenomeles speciosa acted on RAW264.7 cells, cellular viability initially increased and then decreased with the rise in polyphenol concentration [26]. High concentrations of polyphenols could induce oxidative stress, leading to excessive generation of reactive oxygen species. These radicals can damage cell membranes, DNA, and proteins, ultimately causing cell injury and death. Therefore, concentrations of 50 μg/mL and 100 μg/mL were selected for subsequent experiments.

#### 3.6.2. Effects of P−RRTP−FP and P−RRTP−BP on NO Production of LPS−Induced RAW264.7 Cells

**Figure 6:**
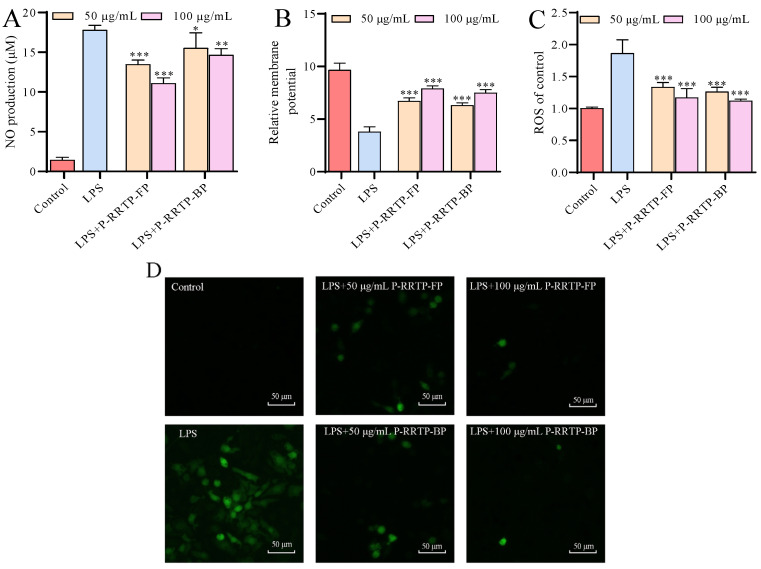
Effects of P−RRPT−FP and P−RRTP−BP on (**A**) LPS−induced nitric oxide (NO) production, (**B**) mitochondrial membrane potential, and (**C**) ROS production in RAW264.7 macrophages. (**D**) Assessment of ROS production through fluorescence microscopy. * *p* < 0.05, ** *p* < 0.01, and *** *p* < 0.001 compared to the LPS−stimulated group. Data are shown as the mean ± standard deviation (*n* = 3).

Upon activation, macrophages can release significant amounts of NO, making NO production a reliable indicator of macrophage activation. As shown in Figure 6A, after treatment with 1 μg/mL of LPS, the release of NO from the cells increased, reaching 12.25 times that of the blank group, indicating the successful establishment of the inflammation model. After being treated with P−RRTP−FP and P−RRTP−BP, the release of NO from cells after LPS treatment significantly decreased and showed a dose−dependent effect, indicating that the two polyphenols have anti−inflammatory effects. Notably, P−RRTP−FP demonstrated more pronounced anti−inflammatory effects compared to P−RRTP−BP. At a dose of 100 μg/mL, P−RRTP−FP showed a 43.33% reduction in NO release compared to the control group, whereas P−RRTP−BP exhibited a decrease of only 17.61%. 

Epigallocatechin was identified as the main polyphenol in P−RRTP−FP, while EA was predominant in P−RRTP−BP. Epigallocatechin, a type of tea polyphenol, has been reported to possess potent anti−inflammatory activity [27]. Conversely, previous studies have indicated that EA exhibits a relatively moderate anti−inflammatory effect [28]. The reduced anti−inflammatory impact of P−RRTP−BP compared to P−RRTP−FP might be attributable to the higher concentration of epigallocatechin in P−RRTP−FP, whereas ellagic acid predominates in P−RRTP−BP.

#### 3.6.3. Effects of P−RRTP−FP and P−RRTP−BP on Mitochondrial Dysfunction in LPS−Induced RAW264.7 Cells

Mitochondria are essential cellular organelles involved in oxidative and energy metabolism. However, due to their unique structure, the mitochondrial DNA in the matrix lacks histone protection, making it susceptible to damage during inflammatory responses, often resulting in a reduction in membrane potential. Therefore, changes in mitochondria can be used as an indicator to reflect the extent of damage caused by inflammation in the body [29]. We assessed the mitochondrial membrane potential of RAW264.7 cells using the JC−1 method. As shown in Figure 6B, compared to the LPS−induced group, the control group maintained a higher level of mitochondrial membrane potential, which was 2.54 times higher than that of the LPS group. The P−RRTP−FP and P−RRTP−BP effectively reversed the negative effects caused by LPS; the high−dose P−RRTP−FP−treated group had a membrane potential 2.07 times higher than that of the LPS group, while the other polyphenol−treated groups exhibited similar effects. Previous studies have also indicated that polyphenols extracted from durian could mitigate mitochondrial polarization [30].

#### 3.6.4. Effects of P−RRTP−FP and P−RRTP−BP on Intracellular ROS Production of LPS−Induced RAW264.7 Cells

The interaction of the cellular immune system with endogenous or exogenous inflammatory stimuli determines the generation of ROS, which causes the hyperactivation of inflammatory responses and leads to tissue damage and oxidative stress. Therefore, ROS activation could act as a significant and adverse participant in abnormal inflammatory diseases. Figure 6C shows the effects of P−RRTP−FP and P−RRTP−BP on intracellular ROS production in LPS−induced RAW264.7 macrophages. The cells stimulated with LPS (1 μg/mL) showed intracellular ROS elevation in comparison to the control group. Compared with the LPS group, pretreatment with P−RRTP−FP and P−RRTP−BP significantly reduced ROS levels in a dose−dependent manner (*p* < 0.05). Particularly noteworthy is that high concentrations of P−RRTP−FP and P−RRTP−BP nearly reversed ROS levels by approximately 37.1% and 40.3%, respectively, compared to the LPS group. A previous study demonstrated that 100 μg/mL of pomegranate peel polyphenols reduced ROS levels by approximately 41.2% compared to the LPS group, reinforcing our findings [31]. Similar results were also observed using fluorescence microscopy (Figure 6D).

#### 3.6.5. Effects of P−RRTP−FP and P−RRTP−BP on LPS−Induced mRNA Expression of Inflammatory Mediators

**Figure 7:**
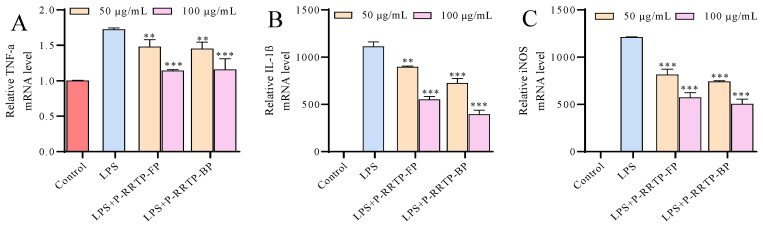
Effects of P−RRTP−FP and P−RRTP−BP on the mRNA expression of pro−inflammatory mediators: (**A**) TNF−α, (**B**) IL−1β, and (**C**) iNOS mRNA expression levels were measured using qRT−PCR; ** *p* < 0.01 and *** *p* < 0.001 compared to the LPS−stimulated group. Data are shown as the mean ± standard deviation (*n* = 3).

The activation of macrophages by LPS prompts the production of various inflammatory enzymes, such as iNOS, and inflammatory cytokines like TNF−α and IL−1β; iNOS serves as a pivotal marker of inflammation, leading to the production of NO [32]. Therefore, we examined the impact of P−RRTP−FP and P−RRTP−BP on the mRNA expression of pro−inflammatory enzymes (iNOS) and cytokines (TNF−α and IL−1β) using qRT−PCR. As shown in Figure 7, the inflammatory response induced by LPS led to an increase in the expression levels of cytokine mRNA. However, treatment with P−RRTP−FP and P−RRTP−BP significantly inhibited the upregulation of gene expression (*p* < 0.05). Meanwhile, the mRNA expression levels of iNOS decreased upon treatment, indicating that P−RRTP−FP and P−RRTP−BP might reduce NO secretion by decreasing iNOS expression.

#### 3.6.6. Effects of P−RRTP−FP and P−RRTP−BP on LPS−Induced NF−κB Activation in RAW264.7 Cells

**Figure 8:**
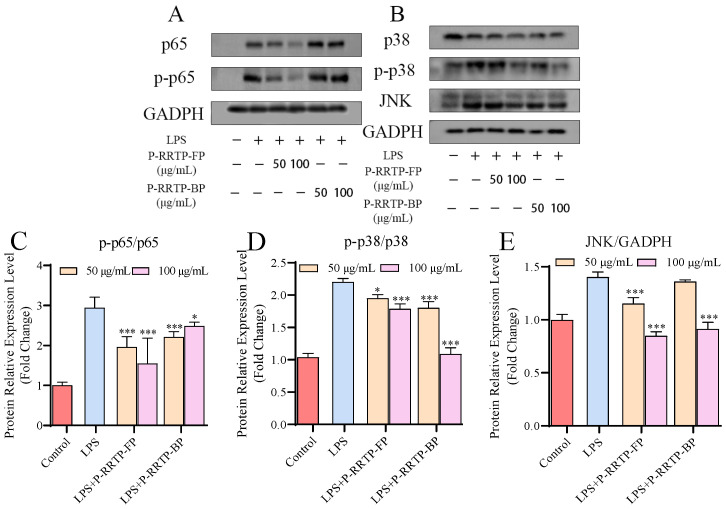
P−RRTP−FP and P−RRTP−BP regulated related signaling pathways in RAW264.7 cells: (**A**,**B**) Western blot of NF−κB and MAPK pathways. The phosphorylation levels of (**C**) p65 and (**D**) p38, and protein expression levels of JNK (**E**). * *p* < 0.05 and *** *p* < 0.001 compared to the LPS−stimulated group. Data are shown as the mean ± standard deviation (*n* = 3).

The NF−κB pathway plays a crucial role in regulating inflammatory processes, as its activation leads to increased expression of pro−inflammatory cytokines, chemoattractant proteins, and their respective receptors [33]. As depicted in Figure 8A,C, there was a significant increase in the phosphorylation of p65 in RAW264.7 cells induced by LPS, compared to the control group. Notably, RAW264.7 cells, following treatment with P−RRTP−FP and P−RRTP−BP, exhibited a significant reduction in the elevated levels of p65 phosphorylation induced by LPS (*p* < 0.05). This finding is consistent with a previous report indicating that phenolic extracts from grape byproducts could decrease NF−κB activation in RAW264.7 cells [34]. In comparison with the P−RRTP−BP group, the superior inhibitory effect on p65 phosphorylation observed in the P−RRTP−FP group may be attributable to the higher contents of epigallocatechin and epicatechin. Previous studies have shown that black tea extract, rich in epigallocatechin, could downregulate the phosphorylation of NF−κB [35]. Based on these results, P−RRTP−FP and P−RRTP−BP regulated the expression of inflammatory mediators and, ultimately, alleviated inflammation by inhibiting the activation of the NF−κB pathway in LPS−induced RAW264.7 cells. The differences in effectiveness may have been due to the specificity of the activities of the different compositions’ components.

### 3.7. Effects of P−RRTP−FP and P−RRTP−BP on LPS−Induced MAPK Activation in RAW264.7 Cells

MAPKs play a critical role in regulating cell growth, differentiation, and the cellular responses to cytokines and stressors. Recent in vitro studies have highlighted the influence of MAPKs, particularly p38 and JNK, on the production of inflammatory mediators in macrophages [36]. To investigate the inhibitory effects of RRTP extracts on inflammatory mediators via the MAP kinase pathway, we assessed the impacts of P−RRTP−FP and P−RRTP−BP on LPS−induced phosphorylation of p38 MAP kinase and the expression of JNK in RAW264.7 macrophages using Western blot analysis. As depicted in Figure 8B,D, pretreatment with 100 μg/mL of P−RRTP−FP and P−RRTP−BP significantly downregulated p38 phosphorylation compared to the LPS group (*p* < 0.05). P−RRTP−BP exhibited notably greater effectiveness than P−RRTP−FP, potentially attributable to its higher abundance of EA. Previous studies have demonstrated that EA can effectively downregulate the expression of p−p38 [37]. JNK is an effective mediator of inflammation. As shown in Figure 8E, treatment with 100 μg/mL P−RRTP−FP and P−RRTP−BP significantly downregulated the expression levels of JNK (*p* < 0.001). The P−RRTP−FP contained substantial amounts of epigallocatechin and epicatechin, while the P−RRTP−BP was rich in EA and epicatechin. Previous research has shown that epigallocatechin and epicatechin, as well as EA, can effectively inhibit the activation of the MAPK pathway [38,39,40]. Therefore, we hypothesize that P−RRTP−FP and P−RRTP−BP inhibit MAPK pathway activation in LPS−induced RAW264.7 macrophages, which may be attributable to epigallocatechin and epicatechin, as well as EA. P−RRTP−FP and P−RRTP−BP might function by suppressing the expression of p−p65, p−p38, and JNK proteins, thereby inhibiting NF−κB and MAPK pathway activation, downregulating the expression levels of TNF−α, IL−1β, and iNOS genes, and consequently reducing the production of NO and ROS, maintaining mitochondrial homeostasis, and thereby exerting anti−inflammatory effects.

## 4. Conclusions

This study highlights the remarkable potential of AB−8 macroporous resin for purifying extracts of both free polyphenols (RRTP−FP) and bound polyphenols (RRTP−BP) from *Rosa roxburghii* Tratt pomace (RRTP), leading to a substantial increase in polyphenol concentration and enhanced antioxidant activity surpassing that of vitamin C. Moreover, the purified RRTP−FP and RRTP−BP (P−RRTP−FP and P−RRTP−BP) exhibited robust anti−inflammatory effects by effectively suppressing NO and ROS production, mitigating cellular membrane potential decline, and significantly downregulating the expression of inflammatory mediators like IL−1β, TNF−α, and iNOS in LPS−stimulated RAW264.7 macrophages. Furthermore, the inhibition of key proteins in the NF−κB and MAPK signaling pathways underscores the mechanisms underlying their anti−inflammatory action. These findings suggest the potential utility of RRTP−derived polyphenols in managing inflammation−related disorders through the development of functional foods or pharmaceuticals. Future research directions may involve in vivo studies to explore the precise therapeutic applications and dosage regimens of RRTP−derived polyphenols for various inflammatory conditions. Additionally, investigating the bioavailability and long−term effects of these compounds could provide further insights into their clinical relevance and safety profile.

## Figures and Tables

**Table 1:** Adsorption and desorption capacities of three macroporous resins for RRTP-FP *.

Resin Types	Adsorption Capacity (mg/g)	Adsorption Rate (%)	Desorption Rate (%)
AB-8	2.33 ± 0.10 ^a^	69.18 ± 2.76 ^a^	94.13 ± 1.92 ^a^
DM130	1.90 ± 0.08 ^b^	57.12 ± 2.11 ^b^	89.19 ± 2.74 ^b^
D101	2.20 ± 0.06 ^c^	59.69 ± 1.18 ^b^	84.03 ± 0.68 ^c^

* Data are expressed as the mean ± standard deviation (*n* = 3). Data in the same column with different letters are significantly different at *p* < 0.05.

**Table 2:** Adsorption and desorption capacities of three macroporous resins for RRTP-BP *.

Resin Types	Adsorption Capacity (mg/g)	Adsorption Rate (%)	Desorption Rate (%)
AB-8	6.67 ± 0.13 ^a^	74.25 ± 1.30 ^a^	97.19 ± 2.62 ^a^
DM130	4.90 ± 0.22 ^b^	62.69 ± 2.46 ^b^	97.37 ± 2.85 ^a^
D101	6.08 ± 0.22 ^c^	47.27 ± 1.04 ^c^	96.04 ± 4.43 ^a^

* Data are expressed as the mean ± standard deviation (*n* = 3). Data in the same column with different letters are significantly different at *p* < 0.05.

**Table 3:** Quantitative results for the major components of RRTP−FP, P−RRTP−FP, P−RRTP−BP, and P−RRTP−BP by HPLC *.

Compounds	Content (mg/g E)			
	RRTP−FP	P−RRTP−FP	RRTP−BP	P−RRTP−BP
Gallic acid	1.42 ± 0.03 ^d^	3.8 ± 0.04 ^c^	17.9 ± 0.43 ^b^	31.63 ± 1.04 ^a^
Protocatechuic acid	2.4 ± 0.03 ^b^	15 ± 0.82 ^a^	1.89 ± 0.05 ^b^	14.8 ± 0.71 ^a^
Ellagic acid	0.92 ± 0.03 ^d^	27.86 ± 0.45 ^b^	23.38 ± 0.44 ^c^	86 ± 0.43 ^a^
Epicatechin	0.77 ± 0.02 ^d^	31.96 ± 0.99 ^b^	7.45 ± 0.11 ^c^	63.27 ± 3.03 ^a^
Epigallocatechin	11.9 ± 0.51 ^b^	160.91 ± 5.35 ^a^	4.36 ± 0.15 ^d^	8.2 ± 0.33 ^c^
Quercetin	1.37 ± 0.06 ^b^	1.57 ± 0.16 ^b^	1.35 ± 0.03 ^b^	14.03 ± 0.21 ^a^

* RRTP: *Rosa roxburghii* Tratt pomace; RRTP−FP: free polyphenols extracted form RRTP; RRTP−BP: bound polyphenols extracted form RRTP; P−RRTP−FP: purified products of RRTP−FP; P−RRTP−BP: purified products of RRTP−BP. Data are expressed as the mean ± standard deviation (*n* = 3). Data in the same row with different letters are significantly different at *p* < 0.05.

## Data Availability

The original contributions presented in the study are included in the article/Supplementary Material, further inquiries can be directed to the corresponding author.

## Supplementary Materials

The following supporting information can be downloaded at https://www.mdpi.com/article/10.3390/foods13132044/s1: Table S1: Primers used for TNF−α, IL−1β, iNOS, and GAPDH. Table S2: Kinetics parameters for RRTP−FP’s adsorption onto the AB−8 resin. Table S3: Kinetics parameters for RRTP−BP’s adsorption onto the AB−8 resin. Table S4: Total phenolic contents of RRTP−FP, RRTP−BP, P−RRTP−FP, and P−RRTP−BP.
