# Supplementary material for: Purification, Characterization, and Anti-Inflammatory Potential of Free and Bound Polyphenols Extracted from Rosa roxburghii Tratt Pomace
Source: Foods. 2024 Jun 27;13(13):2044. doi: 10.3390/foods13132044 (PMC11240960; doi:10.3390/foods13132044)
Supplement: Supplementary file 1 [file foods-13-02044-s001.zip › foods-3038286-supplementary.pdf]

Table S1. Primers used for TNF-α, IL-1β, iNOS, and GAPDH

mRNA	Primer	Sequence (5′–3′)	Size (bp)
TNF-α	Sense	TGTCTCAGCCTCTTCTCATTCC	152
	Antisense	TTTGTGAGTGTGAGGGTCTGG	
IL-1β	Sense	GGGAAACAACAGTGGTCAGG	248
	Antisense	CCATCAGAGGCAAGGAGGA	
iNOS	Sense	ATCCGATTTAGAGTCTTGGTGAA	83
	Antisense	GTGGCTCTGACCCGTGAAG	
GAPDH	Sense	GGTTGTCTCCTGCGACTTCA	183
	Antisense	TGGTCCAGGGTTTCTTACTCC	

Table S2. Kinetic parameters for RRTP-FP adsorption onto the AB-8 resin

Dynamics Models	Kinetic Equations	Parameters
Pseudo-first-order model	$\ln(q_e - q_t) = - 0.32637t + 0.36252$	$k_1 = 0.32637$
		$R^2 = 0.96463$
		q_e = 1.436 mg/g
Pseudo-second-order model	$t/q_t = 0.39353t + 0.71441$	$k_2 = 0.39353$
		$R^2 = 0.99833$
		q_e=2.207 mg/g
Intra-particle diffusion model	$q_t = 0.98432t^{1/2} + 0.09272$	k_p= 0.98432 mg·(g·h)$^{-1/2}$
		$R^2 = 0.97614$
		C= 0.09272 mg/g
	$q_t = 0.12189t^{1/2} + 1.82266$	k_p= 0.12189 mg·(g·h)$^{-1/2}$
		$R^2 = 0.89198$
		C= 1.82266 mg/g
	$q_t = 0.10028t^{1/2} + 1.93999$	k_p= 0.10028 mg·(g·h)$^{-1/2}$
		$R^2 = 0.9842$
		C= 1.93999 mg/g

Table S3. Kinetic parameters for RRTP-BP adsorption onto the AB-8 resin

**Dynamics Models**	**Kinetic Equations**	**Parameters**
Pseudo-first-order model	$\ln(q_e - q_t) = -0.30385t + 1.42905$	k_1= 0.30385
		R^2= 0.97412
		q_e = 4.174mg/g
Pseudo-second-order model	$t/q_t = 0.1295t + 0.14128$	k_2= 0.1295
		R^2= 0.9995
		q_e=7.078 mg/g
Intra-particle diffusion model	$q_t = 2.09645t^{1/2} + 1.86028$	k_p= 2.09645 mg·(g·h)$^{-1/2}$
		R^2= 0.95771
		C= 1.86028 mg/g
	$q_t = 0.59721t^{1/2} + 5.08271$	k_p= 0.59721 mg·(g·h)$^{-1/2}$
		R^2= 0.97534
		C= 5.08271 mg/g
	$q_t = 0.06217t^{1/2} + 6.89306$	k_p= 0.06217 mg·(g·h)$^{-1/2}$
		R^2= 0.98113
		C= 6.89306 mg/g

Table S4. Total phenolic content of RRTP-FP, RRTP-BP, P-RRTP-FP, and P-RRTP-BP *

Samples	Yield (%)	Total phenolic content (mg GAE/g E)
RRTP-FP	23.71±2.80	213.33±8.57
RRTP-BP	2.99±0.28	501.86±4.86
P-RRTP-FP	10.22±1.2	433.79±12.78
P-RRTP-BP	1.07±0.74	833.11±13.92

*RRTP: Rosa roxburghii Tratt pomace; RRTP-FP: free polyphenols extracted form RRTP; RRTP-BP: bound polyphenols extracted form RRTP; P-RRTP-FP: purified products of RRTP-FP; P-RRTP-BP: purified products of RRTP-BP. Data are expressed as mean ± standard deviation (n = 3).
